# Commentaries on Hahnemann’s *Organon*—the Protoplasmic Cell and the Life-Force of Man

**Published:** 1899-08

**Authors:** B. Fincke

**Affiliations:** Brooklyn, N. Y.


### COMMENTARIES ON HAHNEMANN'S *ORGANON*—THE PROTOPLASMIC CELL AND THE LIFE-FORCE OF MAN.

[*Organon* 5th Ed., Secs. 7-17.]

B. Fincke, M. D., Brooklyn, N. Y.

"While there are many scientific men there are few scientific thinkers."
—Drummond, *Ascent of Man.*

The high priest of Cellology has written a pamphlet in 1849, entitled, *The Efforts at Unity in Medicine* (Die Einheitsbestrebungen in der Medicin), in which he said what was not new: "The cell is the simplest form of vital utterance which is represented fully by the thought of life, is the organic unit, the indivisible living thing." "Life is bound to a determined form of the cell, without it it does not appear. The expression of life, therefore, is the formation of the cell, the organization; only the organic is alive." Yet "the cell itself is not from the beginning a cell, it does not stand there all on a sudden, but a series of successive processes presents itself to our eye, the last term of which is the perfected organic unit, the cell." "To our eye" is here meant metaphorically, for nobody ever has watched the formation of the original protoplasmic cell. The analogy which he now cites moves on a sophistical ground. He compares the mother-liquor from which the crystal starts with the blastema or protoplasma from which the cell is formed. In both cases these are physico-chemical processes which furnish the morphological product of the crystal and cell. To be sure life is to him nothing but the physico-chemical process which from the forming crystal that can be watched is applied to the forming cell that no eye can see. Then he says: "The outer plastic act, the organization which we may parallelize with the crystallization, is dependent upon the one preceding it, it can be considered as the necessary result of the properties of the new chemical materials formed by the inner act as the association of these materials to distinct forms based upon their properties." There we have now the favored properties of matter which are to mediate the process of the organization of the cell as well as the crystal. *Principiis obsta!* What are these properties after all? They are handled like legitimate coin in the physico-chemical realm, but can find no application in the doctrine of life or biology. They are the forces of lower life given to matter of multifarious kind. All at once the author finds that "the inner preparatory act, the premorphological motion, is a chemical catalytic act," in order to make the genesis of the cell more comprehensible, that the inner catalytic but, properly speaking, genetic act is not introduced at all by the chemical affinities," which are his properties. He brings in the new conception, catalysis, which belongs to chemics, in order to bring it nearer to the genesis of the cell. But it is a direct contradiction to his former assertion, and he acts like the impartial judge who gave both parties right, only in the reverse, inasmuch as he blames both the chemists and vitalists as being in the wrong, and he finds an exit from the blind alley in which he finds himself in the mechanical perception. But this will not do, because even "the mechanical laws of physics and chemics are not manifest everywhere and at all times, as he says, because they are often latent." And yet, the "processes of life, inasmuch as they occur at mechanical materials (now, what is mechanical materials?), offer in single cases no deviation from the common utterances of the mechanical laws." And yet, "the mechanics of life, the physics and chemics of the vital processes do not contain the fundamental ground of unity." But above the premorphological motion was a chemico-catalytical act. How can that be reconciled? Through a questionable kind of reasoning he comes to the "excitation" (Erregung) as the cause of motion, and since every motion presupposes an excitation, as one is already the sequel of the other, he is urged to assume an original excitation, a creation, and now the endless series of excitation causing motion comes to a sudden end; when he declares the first term, the excitation to be "transcendent because it leads upon a field which contradicts all experience and for which every possibility of a perception of conscious knowledge is missing." Thus the cat walks around the hot porridge, for he cannot escape the conditions of the origin of the first protoplasmic cell which now to him is a mechanical affair resting upon the ordinary physico-chemical mechanics—*i. e.*, making the ordinary properties of the materials contained in the blastema or protoplasma manifest. Now he arrives at the reasonable thought that the excitation of this motion is not this motion itself, and he admits that science cannot answer such questions, for he has no idea of what motion really is. In spite of his admission that "the chemist has not yet succeeded to compose the bodies of the blastema (fibrine, albumine, starch, etc.) from the elements, and the physicist has as yet been unable to form any of these bodies when they were given, into organization outside of the living body," he still falls a prey to the physico-chemical perception with its material premises, because he is wrecked upon the definition of motion. Though, as he says, mechanics and life are not identical, yet in the same breath "life is a peculiar kind of mechanics and the most complicated form at that." Here is the great difference between Hahnemann and the physico-chemical school. Hahnemann, with many of his predecessors and successors, has seen life not only in the organic beings which developed from the original protoplasmic cell, but also in all inorganic matter, and said: "Everything lives." Only everything according to its kind and degree, from the lowest to the highest. Already in the expression "motion" lies the direction from which the scientists *par excellence* hie away to hide their heads in order not to see what they do not want to see. The expression "motion" points to an existence of something acting. The horse which draws the wagon moves, the wagon does not move by itself. The matter is inert and moves only when it is moved. But the matter is kept in its integrity by that which gives it its form, its weight, its affinity for other matter. But, says the thinker of the physico-chemical school, this goes beyond my horizon, *ignoramus* and *ignorabimus* cries another authority, *agnoscimus* still another. Yes, all the great naturalists who have blessed mankind with so many useful and brilliant inventions, admit that they cannot penetrate that board of their own application before their eyes. And thus it happens that the protoplasmic cell is exalted to the dignity of a creator of man though it is only the material in the invisible hand of the Almighty builder. Since our author cannot proceed any further, he, nine years later, in his Cellular Pathology, startles us by the declaration, "Every animal (to which man is reckoned) appears as *a sum of vital units* of which every one carries the full character of life. The character and the unity of life cannot be found at a distinct point of a higher organization—*e. g.*, in the brain of man but only in the distinct constantly acting arrangement which every single element carries in itself. From this it follows that the composition of a larger body always amounts to a kind of social institution where a mass of single existences are intimately related to each other, but so that every element has a special activity for itself, and that every one allows the essential performance to get out of itself though the incitation to such activity is received from other parts." In these words he proclaims the socialistic commune of the cell-government in the organism (which is incompatible with the American system), therefore the direct contrary to his efforts of unity in medicine, viz.: the multiplicity of life in all the organic world.

In the course of time follow the extravagant ideas expressed by other great lights of evolution, one of which, *e. g.*, assigns "the most ancient and simple form of love to the elective affinity of two simple cells." But the latest investigators in biology surpass everything that a rational being can think of. The human creature can think much, but when there is no logical truth in it, what is it good for? The devotees of Christian Science, *e. g.*, say there is no such thing as matter. There is no such thing as individual mind. All is mind, there is no matter, matter is mind. Matter is the denial, not the fact of existence. The opposite of truth is error. Where is here any science, nay, any sense in these utterances? Christ, they say, was imperfect, not having conquered the belief in material life. He never died. There is no resurrection. Where is, then, the Christianity of Christian Science? It is nothing but a nonsensical perversion of thought, and cannot be admitted for a moment to consideration. All the pomposity with which these teachings are preached cannot help their falsity and confusion, and are sophistry, but not philosophy. Another monstrosity of egregious nonsense is that Book of Mormon, which is full of abominations, immorality, and blasphemy, and contradicts what its adherents profess before the American nation. Another scientific thinker says:

"The simplest cell is capable of feeling a stimulus and of adopting means to ends. Only mind can feel and make any adaptive reaction. A cell remembers its experience, and only mind can remember. A piece of protoplasm can do these things, and therefore it is animal. It follows that life is mind. The cells out of which an animal is built are mind-organisms, and the duty of each cell are duties which require mind for their performance. The life of the cell is its mind." This looks like logic, but is only a caricature of it. Because the simple cell of an amœba changes its form of a sac to reach at something and shoots out a projection like a finger, or to change its place, it must have a mind, a soul. What a poor idea the learned man must have of a soul if he could transfer his observation of the simplest cell upon the protoplasmic cell of man without heeding the great intervening chasm separating them? The simple operation of life of a simple-cell-animal on the lowest rung of the organic ladder in relation to its nourishment and motion seduced him to assume the presence of mind, of a soul, understanding, intelligence, memory, will, common and special sensation, consciousness and conscience. Against such an induction, which is nothing but a stretching of a fact to unjustifiable conclusions, a venturous jump into vacancy, science should defend itself; but it does not. If philosophy is the working with conceptions, the first thing to be sure of is the correctness of our conceptions as the premises for our conclusions. But if they are uncertain, confused, and perverted, they can only bring the misery of misunderstanding among those who want to progress in knowledge seeking the light, and are apt to lead weak intellects to disappointment and despair, if not to the insane asylum.

The simple protoplasmic cell which, according to the testimony of the biological students, proceeds first out of the surrounding blastema or protoplasma, which itself again proceeds from materials which no physics, no chemics can produce, and which nevertheless exists in the motherly organ ready to be set in motion after the sexual conception in such a way that from it the first organic formation of the protoplasmic cell proceeds, this at once is declared with great emphasis as the acme of the human organization to which without much ado are assigned all the spiritual activities which it contains, and upon it the glorious hypothesis of evolution is erected which finally points to the origin of man by physico-chemical processes. The good people forget in their zeal that the transcendentality which precedes the formation of that cell and which they have excluded from the precincts of their science as inaccessible to experience, goes through the entire creation, as it ascends from the observations of a series of organic beings from the lowest to the highest, the human being; yea, as it repeats itself in these various stages of creation in the motherly organ in the incarnation of man. Thence comes the misfortune which the materialistic philosophy has brought upon the world, inasmuch as it leads man away from his divine origin, and the constant divine guidance during his life and substitutes fundamentals which instead of being grounded upon the rock of universal life in nature, infused by the Almighty, are built upon the quicksand of the multiplicity of cells. What a violence is done to reason when the primitive protoplasmic cell is made responsible for the total organism! However, all the biological inquirers are unanimous in the fact, that "the first embryonic abodes of the moss and fern and pine or shark and crab and coral polyp, of lizard, leopard, and man are so exactly similar that the highest powers of mind and microscope fail to trace the smallest distinction between them." Now the monstrosity of the contradiction with the well-known fact to the almightiness of the protoplasmic cell, as far as the formation is concerned, is silenced by the introduction of its environment into the argument, from which the subsequent developments to organs and the continued combination among them to the complete organisms are explained. But it is only a disingenuous logical theft in order to escape from the animating thought that behind the protoplasmic cell and its environing blastema is the high potency given by the Eternal, which models the materials in the motherly organ as an unseen sculptor to whom not only the crude stone and clay, but even the elements which compose them, with all their indwelling life-forces are subjected. How should this, to the observer, invisible cell when it enters the fallopian tube, possess the property to move on, to imbibe nourishment, and to grow in certain directions if not the short human understanding like skillful prestidigitator would substitute for the acting high potency his own physico-chemical conceptions, which are good enough for physics and chemics, but not for the biology of man? What arrogance of these brave scientific men who want to impute the highest honor of science to the protoplasmic cell when they place it above Him who has created them with all their acquirements and lovingly holds them in His almighty hand as His children! How can they dare to assign to this simple, primitive cell such a high place which does not belong to it? It does not last long before this same cell is gone forever. It has doubled itself and occupies a larger space than before. It has lost its identity because it is a double body now, and then it continues to divide into embryonic cells, which increase in number and decrease in space till finally they result in a large cell with innumerable small cells inside which are quite different from the one that entered the tube. Now it enters the abode of the motherly organ, which serves as the receptacle for the embryo, to be formed from that cell, and the next appearance presents to the observer a simple line from which gradually the organs of the new being are developed. O Thou wonderful Architect of the Universe, how hast Thou with unsurpassed wisdom undertaken the building up of the future human being; Thou to whom all forces are subject which are given to the ubiquitous, so mysterious, and yet so manifest, life in all things!

Of right the German inquirer says: "Behind the co-operating forces of nature, which aim at one goal, we must recognize a cause—incomprehensible, according to its nature of which we can only say with certainty that it is theological." What an uproar there was in that convention of the physicians and natural philosophers when one of their greatest members declared that science were the propædeutics of religion! How at once he fell from his high estate, being considered as laboring under the infirmities of old age by these aristarchs of science!

If now the further development of the organism proceed by renewed formation of cells, and rebuilding and changing of the cells into organs, it is another logical theft to endow the protoplasmic cell which lost its identity long ago in the subdivision and change in its transit through the tube, with forces which appear in the continually perfecting organs till in the complicated organ of the mind, the brain with its nervous system they harbor the spiritual nature of man. Now, by the trick of attributing already to the original protoplasmic cell the forces which belong to the perfected cellular structure of the brain, they deny just that which makes the unity of the total parts of the body in their multiplicity possible, the life-force which dynamically enables the lowest and highest organs built up from cells to pursue a regular course of action for the existence and benefit of the whole being, so that if any disturbance occurs, from out or inside, there is a possibility to reach it by the homœopathic remedy in lower or higher potency. These investigators, who award to the protoplasmic cell the physiological omnipotence, could just as well make the small part of drug responsible for the whole series of symptoms which can only be obtained by a series of its potencies in its provings, and they could just as well attribute to it also the cure which the homœopathician can make by the administration of a hundred-thousandth or a millionth potency, if he proceeds according to homœopathic principles. How could we, without the Hahnemannian doctrine of the life-force, imagine how a high potency could heal a suffering in the most remote part of the body when we place a few globules moistened with the potency upon the tongue and, *e. g.*, remove the gouty pain in a big toe, or cure a long-standing psoriasis palmaris, or quiet mental excitement, produce sleep or stop blood-poisoning, etc. These scholars who expect so much from the simple protoplasmic cell should first localize the cell or cells which are made responsible for these disturbances of the life-force, and then indicate the means by which the healing remedies can reach them. They cannot do it because they are without the guiding principle of the healing art and science and follow the crudest empiricism and the ever-changing kaleidoscope of pathological opinion. It is clearly out of their range of vision, since they have not the least idea of potentiation, and simply ignore, deny, and ridicule what the homœopathician, through a long series of years and laborious experiments has found out.

Only recently a German professor who is on the road to Homœopathy, expressed his disapproval of the cellular theory in these words: "The physician treats at the bedside not only sick cells, but sick organs. That what he sees and what the patient feels are not changes occurring at the single cells, but the phenomena presented singly or in the totality by the organism deviating from the normal state. The cell belongs to the theoretical science; the organ, the organism to the practice. Of what happens in the pathologically acting cells when we administer medicines we can at most have only ideas, but a well-founded knowledge in this respect is at the present time out of the question, and it cannot well be otherwise on account of the great difficulties opposing the final solution of it. With hypotheses, however, may they be ever so ingeniously thought out and fascinating at the first sight, the medical practice, the therapy in the true sense, is not satisfied."

In order to justify the inexcusable attitude of the allopathic biologists in regard to the protoplasmic cell, their honesty is made their excuse. But what honesty has to do here is not easy to be seen. If one calls his honesty to his aid, to supply his ignorance of scientific knowledge, he enters upon a field with which natural science has nothing to do, morality. He may settle that with himself and let others alone.

How the life-force of the organism is connected with the material parts of it, is no clearer than when the protoplasmic cell gets the ability to introduce the development and perfection of man; and the knowledge is not indispensably necessary for healing. When, however, the unity of the organism, with its multitudes of cells and the organs resulting therefrom, must rationally be acknowledged, since the facts prove it, in the course of human life as well as in its physical and chemical utterances and its spiritual activity, the opinions of the allopathic biologists cannot be held, and must be thrown overboard without mercy. Only then new points of view will appear which presently by the by-ways of bacterianism and the arrogance of commercial chemistry to assume the business of healing are veiled. Just as these allopathic biologists throw the divine influence out of science as transcending their experience, they do also with the life-force of Hahnemann, with his high potencies, and do not even know that they deprive themselves of the only standpoint which they can have in science at all, because they consider Homœopathy as standing outside of all science.

*Ceterum censeo macrodosiam esse detendam.*

